# Postsynthetic Modification of NU-1000 for Designing a Polyoxometalate-Containing Nanocomposite with Enhanced Third-Order Nonlinear Optical Performance

**DOI:** 10.1021/acs.inorgchem.2c02709

**Published:** 2022-11-14

**Authors:** Yangdan Pan, Soheila Sanati, Marzieh Nadafan, Reza Abazari, Junkuo Gao, Alexander M. Kirillov

**Affiliations:** † The Key Laboratory of Advanced Textile Materials and Manufacturing Technology of Ministry of Education, National Engineering Lab for Textile Fiber Materials and Processing Technology, School of Materials Science and Engineering, Zhejiang Sci-Tech University, Hangzhou310018, China; ‡ Department of Chemistry, Faculty of Science, University of Maragheh, 55181-83111Maragheh, Iran; § Department of Physics, Shahid Rajaee Teacher Training University, 16788-15811Tehran, Iran; ∥ Centro de Química Estrutural, Institute of Molecular Sciences, Departamento de Engenharia Química, Instituto Superior Técnico, Universidade de Lisboa, Av. Rovisco Pais, 1049-001Lisbon, Portugal

## Abstract

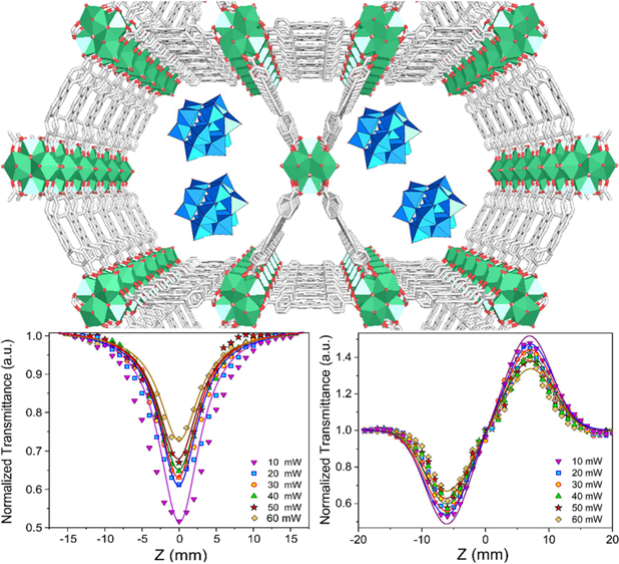

For the advancement of laser technologies and optical engineering, various types of new inorganic and organic materials are emerging. Metal–organic frameworks (MOFs) reveal a promising use in nonlinear optics, given the presence of organic linkers, metal cluster nodes, and possible delocalization of π-electron systems. These properties can be further enhanced by the inclusion of solely inorganic materials such as polyoxometalates as prospective low-cost electron-acceptor species. In this study, a novel hybrid nanocomposite, namely, SiW_12_@NU-1000 composed of SiW_12_ (H_4_SiW_12_O_40_) and Zr-based MOF (NU-1000), was assembled, completely characterized, and thoroughly investigated in terms of its nonlinear optical (NLO) performance. The third-order NLO behavior of the developed system was assessed by *Z*-scan measurements using a 532 nm laser. The effect of two-photon absorption and self-focusing was significant in both NU-1000 and SiW_12_@NU-1000. Experimental studies suggested a much superior NLO performance of SiW_12_@NU-1000 if compared to that of NU-1000, which can be assigned to the charge-energy transfer between SiW_12_ and NU-1000. Negligible light scattering, good stability, and facile postsynthetic fabrication method can promote the applicability of the SiW_12_@NU-1000 nanocomposite for various optoelectronic purposes. This research may thus open new horizons to improve and enhance the NLO performance of MOF-based materials through π-electron delocalization and compositing metal–organic networks with inorganic molecules as electron acceptors, paving the way for the generation of novel types of hybrid materials for prospective NLO applications.

## Abstract 2

A new SiW_12_@NU-1000 nanocomposite was prepared via a postsynthetic modification of the NU-1000 framework with SiW_12_ (H_4_SiW_12_O_40_) polyoxometalate. After complete characterization, the NLO performance of the obtained hybrid composite was studied by the *Z*-scan technique using a Ng:YAG laser at 532 nm, revealing a significant enhancement in the NLO performance of NU-1000 upon the inclusion of polyoxometalate. This work broadens the application of MOFs as promising matrices for hybrid nanomaterials with remarkable NLO properties.

## 1 Introduction

The third-order NLO (nonlinear optical) materials have attracted attention in various fields including harmonic generators, electro-optical signal processing, optical limiting and communications, saturable absorbers, lasers, optical switching, ultrafast photonics, and two-photon photodynamic therapy.^1−3^ In this regard, the development of novel third-order NLO materials with improved performance is of crucial significance.^4,5^ A number of strategies have been adopted to boost the third-order NLO susceptibility, with examples including the introduction of additional organic moieties with delocalization of electrons,^6,7^ semiconductor doping of glasses,^8,9^ and application of metallic nanoparticles to enhance surface plasmons.^10,11^ Despite a long optical response time (pico-nanoseconds), the highest third-order NLO susceptibility (χ^(3)^(ω)) is typically observed for inorganic NLO materials.^9,12,13^ However, the optical response time of polymeric and π-conjugated organic NLO materials lies in the femtosecond range in spite of their lower χ^(3)^(ω) values.^14−16^ Numerous applications of NLO materials have motivated the research toward deep understanding of possible relationships between optical signals and chemical structures, ultimately aiming to find novel substances with a supreme nonlinear optical performance.

**Scheme 1:**
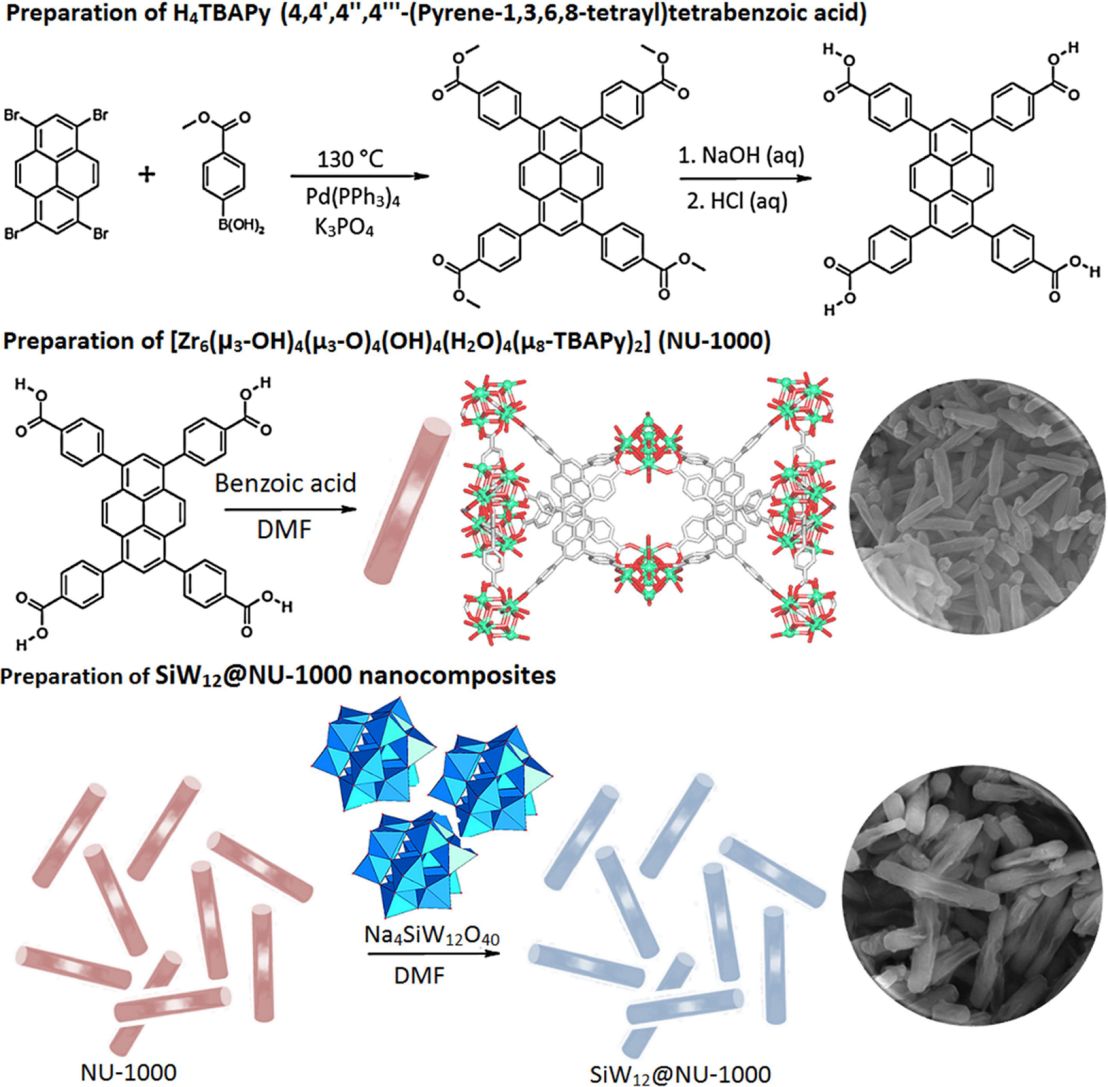
Schematic Depiction of the Preparation of the SiW_12_@NU-1000 Nanocomposite

As a prominent class of crystalline compounds, metal–organic frameworks (MOFs) feature porous structures constructed from metal cations or clusters and organic linking ligands.^17−20^ This class of materials has attracted a colossal attention in many fields, owing to their structural and chemical tunability, high surface area, controllable morphologies, multiple active sites, adjustable porous structures, and variable chemical functionalities.^21−25^ Because MOFs can feature π bonding in their structures, the electron transfer between the organic ligands and metals can significantly enhance the NLO effect.^26−29^ To further improve the NLO performance of these materials, the charge transfer can be tuned by the introduction of strong electron-acceptor/donor groups so that the energy gap can decline by reinforcing the resulting conjugated systems.^30−34^ Among a variety of MOFs, NU-1000 ([Zr_6_(μ_3_-OH)_4_(μ_3_-O)_4_(OH)_4_(H_2_O)_4_(μ_8_-TBAPy)_2_], Scheme 1) has been regarded as an ideal NLO material because of a diversity of features, which include good stability, high size of pores for postsynthetic modifications, tunable electronic structure with π-conjugated organic linkers, large NLO coefficient, and narrow linear adsorption.^35−38^ The application of MOFs in the NLO field is, however, accompanied by several limitations such as insufficient thermostability and optical transparency, which can potentially be overcome by blending MOFs with other materials to generate hybrid composites.^39,40^

However, the NLO performance of MOF composites has been rarely investigated. Given the presence of pores and large voids in the structures of MOFs, various species can be incorporated into frameworks to improve the NLO behavior. Polyoxometalates (POMs) are an example of stable^41−43^ and low-cost inorganic metal-oxygen clusters, which can be considered as ideal acceptors of electrons from organic moieties, particularly due to elevated metal content with high oxidation states. Some prior studies have indicated a proper NLO performance of POMs, which can be attributed to the presence of clusters with ideal electron-accepting capability.^44−46^ Electron transfer is a vital process in the determination of the NLO behavior of materials.^47,48^ An interesting example concerns a significant enhancement in the NLO properties of a composite comprising a planar binuclear Co-phthalocyanine and a polyoxometalate anion, [SiW_12_O_40_]^4–^.^49^

With a motivation based on the abovementioned explanations, the principal objectives of this work consist in (i) the assembly of a hybrid nanocomposite between NU-1000 and SiW_12_ (H_4_SiW_12_O_40_) polyoxometalate, and (ii) the characterization and detailed investigation of the NLO behavior of the generated SiW_12_@NU-1000 nanomaterial. Hence, here, we report the first example of the NU-1000-based nanocomposite (SiW_12_@NU-1000) that bears POM and features a remarkable nonlinear optical performance. Following the analysis of literature data, the present work also opens up the application of hybrid MOF-POM composites in the NLO field.

## 2 Experimental Section

### 2.1 Preparation of the SiW_12_@NU-1000 Nanocomposite

NU-1000 was obtained according to a previous report (see the SI for details).^50^ The stages for the synthesis of NU-1000 and SiW_12_@NU-1000 nanocomposites are illustrated in Scheme 1. Briefly, 0.2 g of NU-1000 and 0.08 g of H_4_SiW_12_O_40_·*x*H_2_O (Aldrich, 99.8%) were placed into a glass bottle (200 mL volume), followed by the addition of 100 mL of dimethylformamide (DMF), 5 mL of EtOH, and ultrasonic treatment for 15 min. The obtained mixture was then kept reacting at ∼115 °C for 12 h. At the end of the reaction, the formed solid product was isolated and washed with DMF and EtOH four times and dried in an oven at 60 °C to produce the SiW_12_@NU-1000 nanocomposite with a molar ratio of ∼1:3 between its two components. For equipment used for the characterization of SiW_12_@NU-1000 and investigation of its NLO properties, see the Supporting Information (SI).

## 3 Results and Discussion

### 3.1 Characterization of SiW_12_@NU-1000

**Figure 1:**
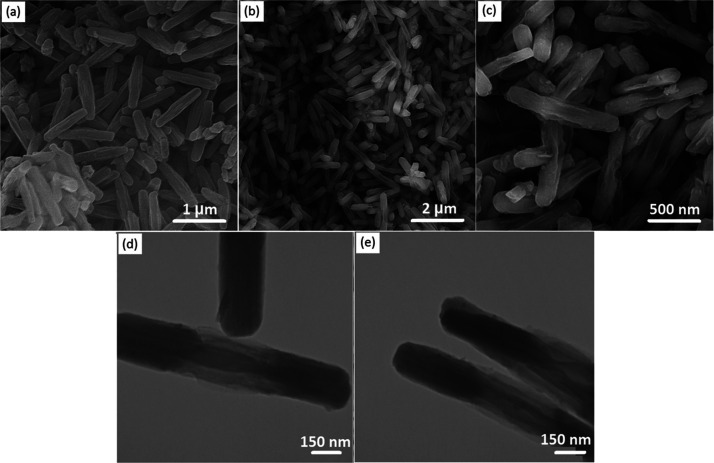
FE-SEM images of NU-1000 (a) and SiW_12_@NU-1000 nanocomposites in two different resolutions (b,c) and TEM images of NU-1000 (d) and SiW_12_@NU-1000 nanocomposite (e).

**Figure 2:**
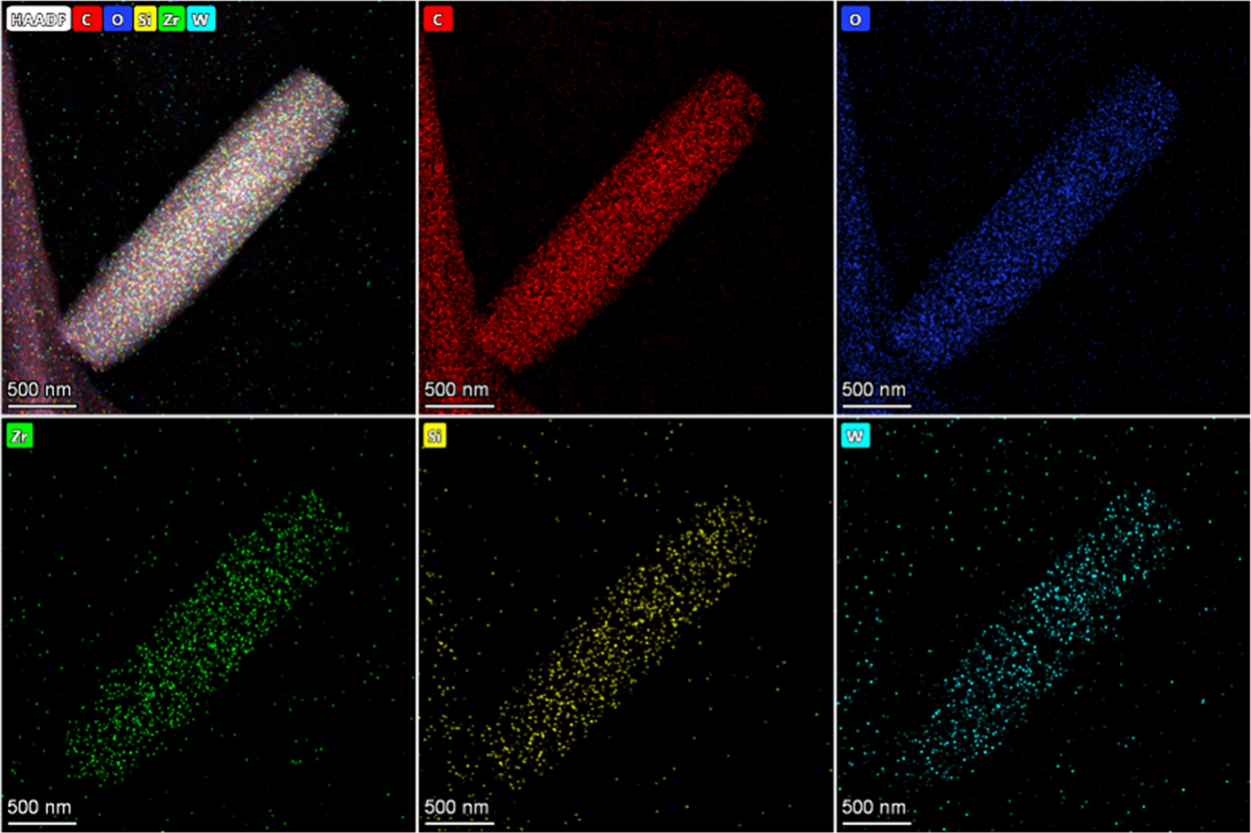
Elemental mapping of the SiW_12_@NU-1000 nanocomposite.

Field emission scanning electron microscopy (FE-SEM) and transmission electron microscopy (TEM) were used for morphological characterization. As illustrated in Figure 1a,d, the synthesized NU-1000 precursor possesses a uniform rodlike morphology with a controlled length of ∼1 μm. Figure 1b,c represent the FE-SEM images of the SiW_12_@NU-1000 composite in two different resolutions, revealing a rodlike morphology similar to that of the NU-1000 precursor. The TEM image (Figure 1e) shows the size of the SiW_12_@NU-1000 composite with a diameter of ∼150 nm and a length of ∼1.5 μm. Also, the elemental mappings of SiW_12_@NU-1000 were conducted (Figure 2), disclosing a uniform distribution of C, O, Zr, Si, and W and demonstrating successful incorporation of SiW_12_ into NU-1000. Besides, the Energy-dispersive X-ray spectroscopy results (three-point average) showed that the contents of C, O, Zr, Si, and W elements are 34.59, 43.13, 17.05, 0.84, and 3.72 wt %, respectively. A relatively low quantity of Si and W can be explained by the presence of SiW_12_ in the pores of NU-1000.

**Figure 3:**
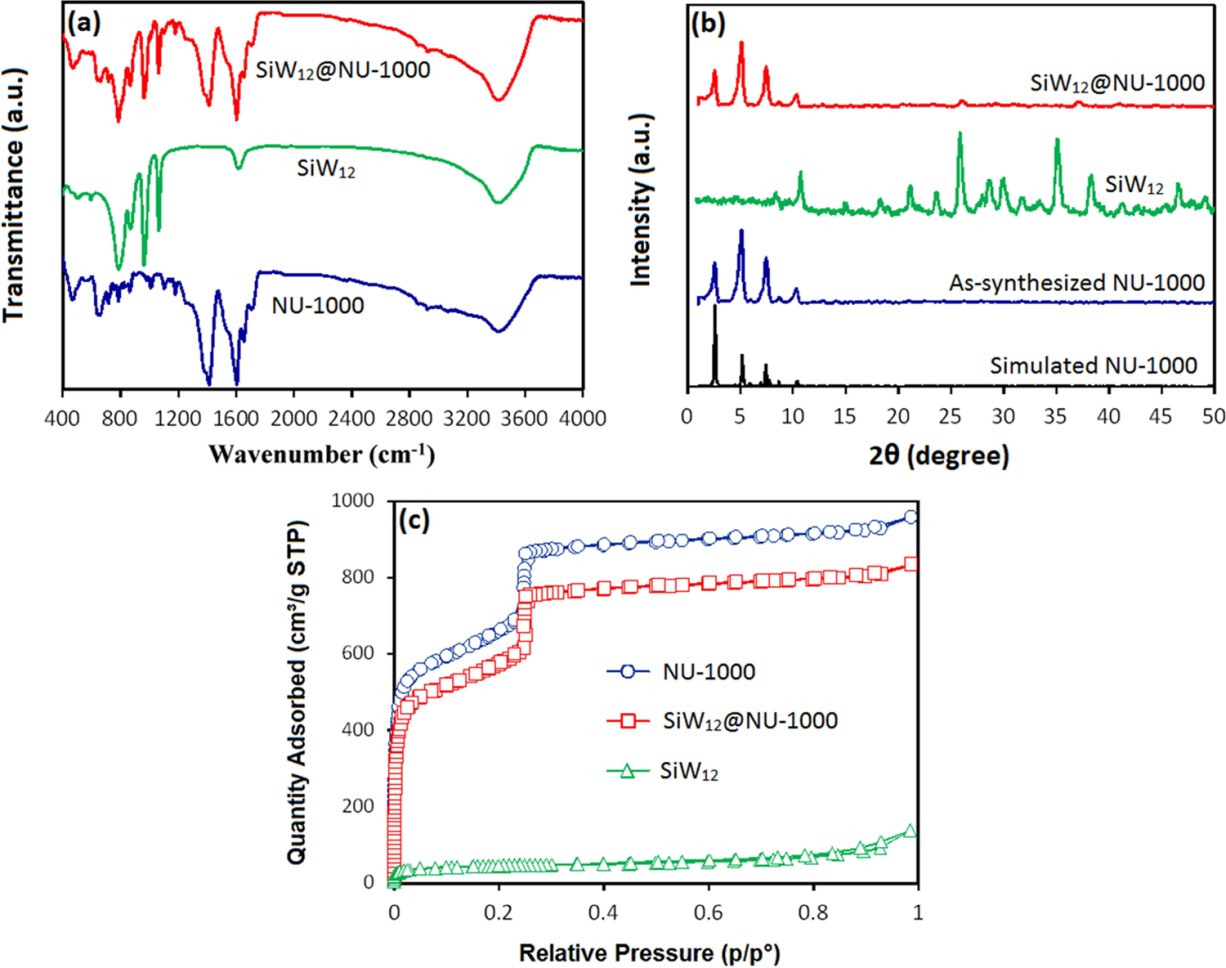
(a) FT-IR spectra, (b) PXRD patterns, and (c) N_2_ adsorption–desorption isotherms of studied materials.

The Fourier transform infrared (FT-IR) spectra of the precursors (SiW_12_, NU-1000) and nanocomposite (SiW_12_@NU-1000) are represented in Figure 3a. In the FT-IR spectrum of SiW_12_@NU-1000, the broad bands with some shoulders with maxima at 1600 and 1478 cm^–1^ correspond to the ν_as_ and ν_s_ vibrations of carboxylate groups and the ν(C=C) vibrations of aromatic rings of the linker in NU-1000.^20^ In SiW_12_@NU-1000, the bands in the range of 600–1100 cm^–1^ are in agreement with those of the SiW_12_ sample. The bands at 1095, 950, and 800 cm^–1^ are ascribed to stretching vibration modes of Si–O, W=O, and W–O–W, respectively.^51,52^ These results further confirm that the SiW_12_@NU-1000 composite was successfully synthesized.

The powder X-ray diffraction (PXRD) patterns of NU-1000, SiW_12_, and SiW_12_@NU-1000 are shown in Figure 3b. The diffraction peaks of the as-synthesized NU-1000 perfectly match those simulated from the crystal structure of this compound, thus confirming the structure and purity of the obtained sample. In the range of 2–12°, the SiW_12_@NU-1000 composite reveals the diffraction peaks similar to those of the as-synthesized NU-1000, thus confirming that the crystalline structure of NU-1000 is retained after the SiW_12_ incorporation. The SiW_12_ diffraction peaks in the PXRD pattern of SiW_12_@NU-1000 are not visible as POM moieties occupy the pores of NU-1000 and are not present on the surface of MOF. These results are in agreement with the related literature data.^53−57^ The characterization by PXRD can also be affected by the crystallinity decline of SiW_12_ when incorporated into SiW_12_@NU-1000. However, the diffraction peaks of both components were observed in the PXRD pattern of the physical mixture of SiW_12_ and NU-1000 (Figure S1, SI). In addition, it is clear from the SEM images and elemental mapping (Figures S2 and S3) that in the physical mixture of H_4_SiW_12_O_40_·*x*H_2_O with NU-1000 (1:3) obtained by grinding, SiW_12_ stays on the surface of NU-1000 and its morphology is completely different if compared with the nanocomposite sample. Thus, because POM is not seen on the composite’s surface, it should be present inside the pores. In the PXRD pattern, there is a weak intensity peak at ∼25.5° that corresponds to SiW_12_, which further supports the incorporation of SiW_12_ into SiW_12_@NU-1000. The nitrogen adsorption–desorption isotherms (Figure 3c) were used to investigate the surface area, microporous volume, and total pore volume of NU-1000, SiW_12_, and SiW_12_@NU-1000. An obvious phenomenon can be observed for a porous composite material, wherein the N_2_ absorption by SiW_12_@NU-1000 is slightly below that of NU-1000 and significantly superior if compared to SiW_12_. A decrease in the N_2_ absorption by SiW_12_@NU-1000 is due to a blockage of the pores of NU-1000 that is caused by substantial incorporation of SiW_12_ (up to ∼30 mol %). The pore sizes of NU-1000 are 3.3 and 1.3 nm,^58^ while the maximum size of SiW_12_ is 1.043 nm (CCDC: 903574), thus enabling a good fitting of SiW_12_ into the pores of NU-1000. Besides, the pore volume of NU-1000 (1.65 cm^3^ g^–1^) decreases in the composite (1.29 cm^3^ g^–1^). Such a 22% decrease in the pore volume confirms the encapsulation of POM in the pores of NU-1000. Any significant structural change in NU-1000 is not observed after loading it with SiW_12_.

Also, we synthesized a sample of NU-1000 according to the protocol of composite formation but in the absence of SiW_12_. As shown in Figure S4, NU-1000’s surface area essentially remained unaltered (2137 vs 2135 m^2^ g^–1^) and the solvent and temperature had no effect on NU-1000. Therefore, under the conditions of nanocomposite synthesis, there is no decomposition of NU-1000 or appreciable change in its surface area. In addition, after the synthesis of the composite in the presence of SiW_12_, the diffraction peaks of NU-1000 are preserved (Figure 3b). Thus, a slight decrease in the surface area of SiW_12_@NU-1000 if compared to the parent MOF is related to the presence of SiW_12_.

### 3.2 NLO Properties

The *Z*-scan method has been widely employed to explore the NLO (nonlinear optical) behavior of materials.^59^ The OA (open-aperture) and CA (closed-aperture) modes were used to perform the *Z*-scan analyses with a previously outlined setup and then to measure NLO.^60,61^ Overall, a constant wave Ng:YAG DPSS laser (diode-pumped solid-state laser) having a 532 nm wavelength was used as the light source, and the beams were concentrated utilizing the 19 cm lens. To scan the samples, a transition stage was monitored by a PC with the ability to transfer to the positive and negative sides of the *Z* = 0 axis in every scan. Preparation of samples was initially performed as a 0.01 mM dispersion in DMF, followed by placing the obtained samples in a 1 mm long quartz cell.

**Figure 4:**
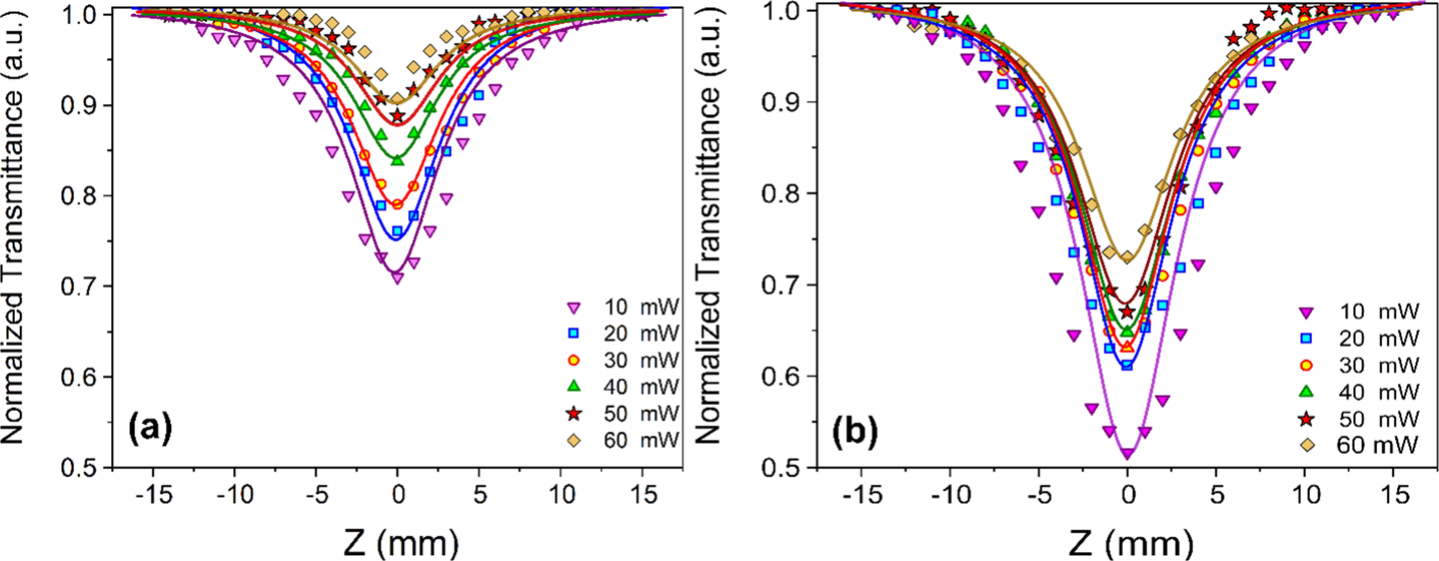
OA curves for (a) NU-1000 and (b) SiW_12_@NU-1000 under various laser incident power values in DMF.

As supported by the experimental evidence, NU-1000 and SiW_12_@NU-1000 perform extremely well as NLO materials upon the use of an uncomplicated but effective single-beam *Z*-scan technique supported by constant wave or CW (continuous wave). The above technique was utilized to determine the NLO coefficients for NU-1000 and SiW_12_@NU-1000, considering different incident laser power values and a constant wave of the TEM_00_ Gaussian mode of the Nd:YAG laser. An example of the *Z*-scan OA mode is represented in Figure 4 for the prepared samples at different incident power values. Eq 1 was used to fit the experimental data and estimate the nonlinear optical absorption (NLA) coefficient, as shown in Table 1.^5,62^
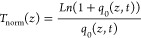
1

**Table 1:** NLO Parameters of NU-1000 and SiW_12_@NU-1000 at Different Power Values of the Laser

sample	*P*_0_ (mW)	α (cm^–1^)	*L*_eff_ (mm)		β × 10^–3^ (cm/W)
NU-1000	10	9.78	0.63	22.3	21.3
	20	8.47	0.67	9.09	10.1
	30	8.26	0.68	7.43	5.95
	40	8.43	0.67	4.35	3.41
	50	9.02	0.65	3.17	2.37
	60	10.7	0.61	2.53	1.92
SiW_12_@NU-1000	10	8.51	0.67	41.8	23.6
	20	7.91	0.69	27.2	13.9
	30	8.05	0.68	20.1	9.73
	40	8.15	0.68	19.2	7.79
	50	8.60	0.67	19.0	6.30
	60	8.10	0.68	18.8	5.46

**Figure 5:**
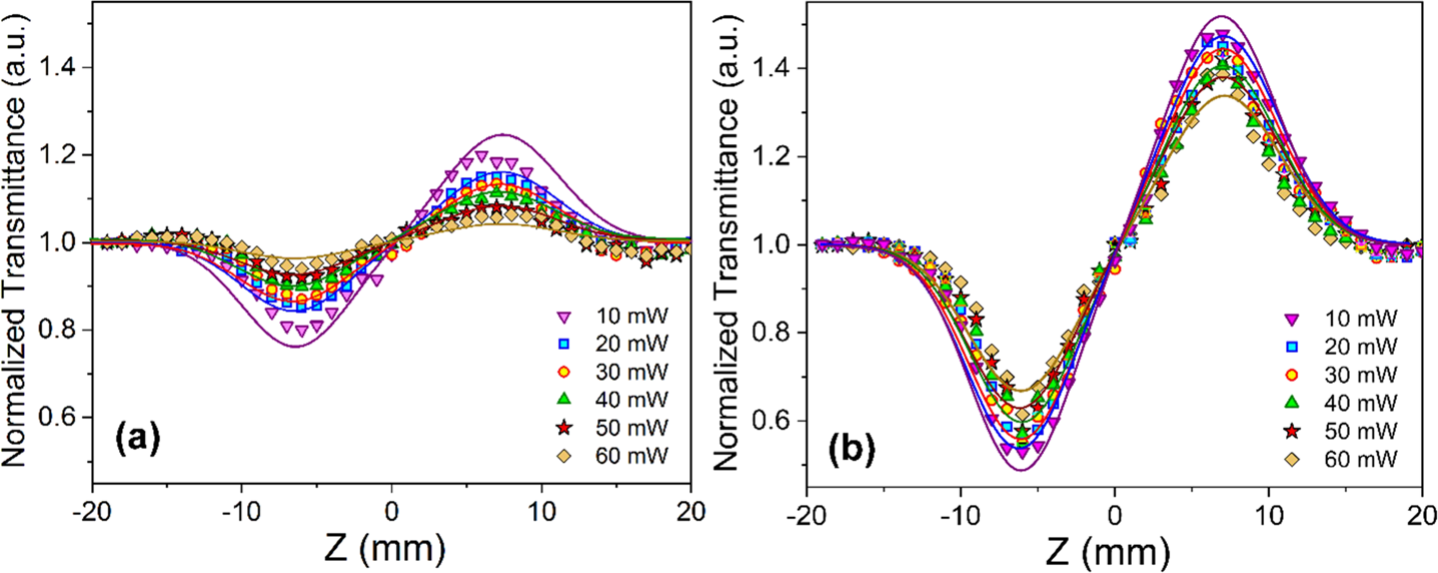
CA curves for NU-1000 and SiW_12_@NU-1000 under various laser incident power values in DMF.

Herein, *q*_0_(*z*, *t*) = β*I*_0_*L*_eff_/(1 + *z*^2^/*z*_R_^2^) and *L*_eff_ = (1 – e^–α_0_L^)/α_0_ denote the sample thickness. Besides, *L* and α_0_ are the path length and the linear absorption (LA) coefficient, respectively, while *z*_R_ = *k*ω_0_^2^/2 and *k* = 2π/λ represent the beam diffraction range and the wave vector, respectively. Finally, λ and *I*_0_ show the laser wavelength and excitation intensity at *z* = 0, respectively. The relation *I*_0_ = 2*P*_0_/πω_0_^2^ was utilized to calculate the value of *I*_0_. The NLA coefficient calculations led to totally negative values, confirming that 2-photon absorption (2PA) responses were present. Furthermore, a decrease in the laser incident power resulted in a deeper valley, revealing an increase in the 2PA response.^63^ The theoretical fitting of the experimental results is presented by solid lines in Figure 5. Different factors, including the functional group types of the ligands, the organic linker configuration in the crystal, and the MOF porous structure contribute significantly to the outcomes of NLO.^64^

The CA/OA *Z*-scan transmission curves for NU-1000 and SiW_12_@NU-1000 under various laser incident power values are shown in Figure 5. For the peak-valley separation of >1.7 *z*_R_ (2.2 *z*_R_), there is evidence for the nonlinear state.^61^eq 2 was used to calculate  (nonlinear refractive index, NLR) with the value of Δ*T*_p – v_.
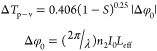
2

In this equation, the value of *S* = 0.28 represents a linear transmittance of the aperture estimated by *S* = 1 – exp (( – 2*r*_a_^2^)/(ω_a_^2^)). Here, ω_a_ and *r*_a_ indicate beam and aperture radii, respectively. When the normal transmittance (*T*_V_) has the minimum value, there is evidence on the downward trend, corresponding to a deeper valley due to higher intensities. On the other hand, the maximum values of normal transmittance (*T*_P_) demonstrate an upward tendency. As the laser incident power declines, an increase is observed in the values of the nonlinear refractive index (NLR index).

The changes in the *n*_2_ (NLR index) and β (nonlinear adsorption coefficient) were 2.53–22.3 cm^2^/W and 2.12–4.99 cm/W for NU-1000 and 18.8–41.8 cm^2^/W and 1.11–4.93 cm/W for SiW_12_@NU-1000, respectively. More significant differences in transmittance would lead to higher values of the NLR index and nonlinear optical response (Table 1). The positive NLR (self-focusing effects) can be supported by the relative values of Δ*T*_p – v_ for the NU-1000 and SiW_12_@NU-1000. Besides, *n*_2_ has a value equal to 10^–8^ cm^2^/W (Table 1).

As shown in Figure 4, NU-1000 and SiW_12_@NU-1000 feature a considerable 2PA under the open-aperture configuration, which can be extensively used to protect optical sensors. A 1.1–2.8-fold improvement can be observed in β values of SiW_12_@NU-1000 at *P*_0_ of 10–60 mW in comparison to the nonlinear optical characteristics of NU-1000. On the other hand, there was a 1.9–7.4-fold improvement in the values of the NLR index (*n*_2_).

The current work aimed to provide useful information on the structure–property relationships in MOF-POM hybrids, which would foster their exploration as novel nonlinear optical materials with greater hyperpolarizability. Because metal clusters contain delocalized dπ–pπ and conjugated dπ–dπ arrangements, they can perform well as nonlinear optical materials.^65,66^ Besides, if metal–ligand and ligand–metal charge transfers are introduced, the nonlinear optical characteristics of these clusters can be improved.^67−69^ The π-electron cloud delocalization mainly gives rise to the nonlinear optical characteristics even though a variety of procedures are applicable for the promotion of such characteristics in metal clusters.^70,71^ Metal ions and organic ligands that construct the skeleton of MOFs are both of great importance in the NLO characteristics of NU-1000 as they primarily lead to the delocalization in metal clusters.

Following the analysis of prior data (Table S1), the remarkable NLO properties of SiW_12_@NU-1000 can justify its prospective applications. As indicated by Hassan et al., significant NLO characteristics were observed in POM-porphyrin hybrids.^72^ According to Hou et al., metal ions and ligands contribute to nonlinear optical materials, given their conjugated dπ–dπ and delocalized dπ–pπ arrangements.^73^ It is also possible to alter the strength of the NLO characteristics by enhancing the metal ion-ligand π-back-donating and creating more extended π-electron systems.^73^ Jia et al. indicated that metals with different radii can have various application prospects in third-order nonlinear materials.^74^ Zhang et al. reported the NLO properties of fulvalene Ru_2_-linked POM, in which the NLO response is enhanced by introducing an electron-donating group (NH_2_).^75^ The contribution of heavy-metal ions to the third-order NLO characteristics is very considerable in metal clusters, permitting more allowed electronic transitions because of the introduction of additional sublevels in the energy hierarchy, thus generating greater NLO effects.^76^ Another factor that can alter the NLO properties of hybrid MOF-POM materials concerns linker ligands used in the construction of MOFs. These linkers can be decorated with functional groups^77,78^ or bear additional aromatic rings to improve the electron density, subsequently enhancing an electron transfer.^79^

According to the *Z*-scan curves, polyoxometalates show insignificant NLO absorption and refractive characteristics under the experimental conditions similar to the present study.^80^ Polyoxometalates are stable and generally soluble inorganic metal-oxygen clusters, the introduction of which into, for example, SiW_12_@NU-1000, enhances electron-accepting properties because of elevated oxidation states of the transition metal atoms within the clusters. It is theoretically anticipated that the nonlinear optical activities can be improved through charge transfer interactions in appropriate donating–accepting complexes.^81^ POM clusters can accept charge from organic electron donors (OEDs). NU-1000 can be among such donors with an excellent NLO behavior because of extensively 2D delocalized π-electrons, allowing to consider this type of compounds as suitable alternatives for optical switching and limiting applications.

According to both theoretical and experimental evidence,^82,83^ charge and/or energy transfer are observed during the formation of donating–accepting systems between polyoxometalates and MOFs, enhancing or tuning the NLO responses. Also, Al-Yasari et al. confirmed that the integration of conjugated ligands into POM increased electron delocalization and effectively improved the NLO performance.^84^ Therefore, organic linkers (e.g., H_4_TBAPy) with conjugated structures are suitable candidates for third-order NLO applications because of their large electron polarizability. Hence, the main contributing factor in enhancing the NLO responses in the hybrid SiW_12_@NU-1000 nanocomposite can be the NU-1000-to-POM charge transfer. The present study represents the first case when POM and NU-1000 are combined within a new composite material to enhance the NLO characteristics.

## 4 Conclusions

In this research, a new SiW_12_@NU-1000 nanocomposite was prepared by hydrothermal method via a postsynthetic modification of the NU-1000 framework with a water-soluble SiW_12_ (H_4_SiW_12_O_40_) polyoxometalate. After complete characterization, the NLO performance of the obtained hybrid MOF-POM composite was investigated by the *Z*-scan method using a Ng:YAG DPSS laser at 532 nm. The obtained data indicate a significant enhancement in the NLO performance of NU-1000 upon an inclusion of POM. Self-focusing effect and 2PA were also observed for both NU-1000 and SiW_12_@NU-1000 samples. The effect of third-order NLO can be modified by regulating the power of the laser. The π-electron cloud delocalization is responsible for the NLO behavior of NU-1000. The important point is that the NLO performance of MOFs can be significantly improved by the incorporation of POM because of the energy transfer between NU-1000 and SiW_12_ as a result of laser collision at various power values. The present research represents a unique investigation of a hybrid material based on NU-1000 and POM in terms of NLO properties. The results of this research can broaden our insight into the design and assembly of advanced MOF-based materials with promising NLO behavior.

## Supporting Information Available

Materials and methods; synthesis of H_4_TBAPy and NU-1000; additional figures with PXRD, SEM, element mapping, and BET (Figures S1–S4); and comparison of NLO materials (Table S1) (PDF)
