# Supplementary material for: Postsynthetic Modification of NU-1000 for Designing a Polyoxometalate-Containing Nanocomposite with Enhanced Third-Order Nonlinear Optical Performance
Source: Inorg Chem. 2022 Nov 14;61(47):18873–82. doi: 10.1021/acs.inorgchem.2c02709 (PMC9775467; doi:10.1021/acs.inorgchem.2c02709)
Supplement: Supplementary file 1 — ic2c02709_si_001.pdf [file ic2c02709_si_001.pdf]

Supporting Information (SI)

Post-Synthetic Modification of NU-1000 for Designing a Polyoxometalate Containing Nanocomposite with Enhanced Third-Order Nonlinear Optical Performance

Yangdan Pan,[†1] Soheila Sanati,[‡1] Marzieh Nadafan,[ǁ] Reza Abazari,[‡,]* Junkuo Gao,[†,]* Alexander M. Kirillov[⊥,]*

[†]*The Key Laboratory of Advanced Textile Materials and Manufacturing Technology of Ministry of Education, National Engineering Lab for Textile Fiber Materials and Processing Technology, School of Materials Science and Engineering, Zhejiang Sci-Tech University, Hangzhou 310018, China*

[‡]*Department of Chemistry, Faculty of Science, University of Maragheh, P.O. Box 55181-83111, Maragheh, Iran*

[ǁ]*Department of Physics, Shahid Rajaee Teacher Training University, P. O. Box 16788-15811, Tehran, Iran*

[⊥]*Centro de Química Estrutural, Institute of Molecular Sciences, Departamento de Engenharia Química, Instituto Superior Técnico, Universidade de Lisboa, Av. Rovisco Pais, 1049-001 Lisbon, Portugal*

[1]*These authors contributed equally to this work*

---

*E-mail: reza.abazari@maragheh.ac.ir (R. Abazari); jkgao@zstu.edu.cn (J. Gao); kirillov@tecnico.ulisboa.pt (A.M. Kirillov).

Materials. All compounds and solvents: 4-(methoxycarbonyl)phenyl)boronic acid (Merck, 98%), 1,3,6,8-tetrabromopyrene (Aldrich, 97%), tetrakis(triphenylphosphine) palladium(0) (Strem Chemicals, 99%), $ZrCl_4$ (Aldrich, 99.5%), tungstosilicic acid hydrate ($H_4[Si(W_3O_{10})_4]\cdot xH_2O$, Aldrich, 99%), K_3PO_4 (Aldrich), benzoic acid (Aldrich, 99.5%), ethanol (EtOH), methanol (MeOH), hydrochloric acid (HCl, Aldrich, 37%) and N,N-dimethylformamide (DMF, Merck) were of analytical grade and used without further purification.

General methods. The FE-SEM images were obtained on a Hitachi S-1460 instrument with 15 kV accelerating voltage. Prior to the FE-SEM examination, the samples were diluted with ethanol, dried on a silica wafer, and sputter-coated by gold. X-ray powder diffraction (PXRD) measurements were performed using a Philips X'pert diffractometer with a monochromated Cu-Kα (λ = 1.54056 Å) radiation. The N_2 adsorption/desorption isotherms were measured at 77 K using a Micromeritics ASAP 2020 analyzer. The specific surface area was calculated by the Brunauer-Emmett-Teller (BET) method. The FT-IR spectra were obtained over the 400–4000 cm^{-1} range on a Shimadzu FT-IR spectrometer (model Prestige 21) using the KBr disks of the samples. Ultrasonication was carried out in a SONICA-2200 EP ultrasonic bath with 40 kHz frequency.

NLO properties. A home-made Z-scan system was employed to evaluate the NLO features of samples at 532 nm. The laser source (Ng:YAG DPSS laser: diode-pumped solid state laser) was focused on the sample with a lens (190 mm focal length). The beam waist radius, ω_0, at the beam focus was varied from 32 to 52 µm. The sample was fixed on a high-accuracy translation stage which moved 0.2 mm at each step. An intensity of the transmitted light was surveyed using a detector.

Synthesis of H_4TBAPy. NU-1000 was synthesized in compliance with the reported processes.[S1] Typically, a mixture of 1,3,6,8-tetrabromopyrene (0.500 g; 0.97 mmol), 4-(methoxycarbonyl)phenyl) boronic acid (1.040 g; 5.80 mmol), potassium tribasic phosphate (1.100 g; 5.30 mmol), and tetrakis(triphenylphosphine)palladium(0) (0.030 g; 0.026 mmol) was loaded and capped in dry dioxane (20 mL) (in a glovebox) into a 20 mL microwave vial (Biotage). Next, the mixture was shaken in an oil bath at 130 °C for 72 h under argon atmosphere. Then, the reaction mixture was dried and washed with water to remove the solid residue and inorganic salt. Moreover, chloroform (three times by 50 mL) was utilized to extract the insoluble substance, and the extract was dried with the magnesium sulfate. Then, the solvent volume reduction under vacuum was performed. Finally, the obtained residue was boiled in tetrahydrofuran for 2 h followed by filtration of the product. The synthesis provided 0.58 g of 1,3,6,8-tetrakis(4-(methoxycarbonyl)phenyl)pyrene (82% yield).

In the next stage, the solution consisting of 1.5 g (37.5 mmol) NaOH in 100 mL THF/water (ratio 1:1) mixture was added to a 250-mL round bottom flask containing 0.58 g (0.78 mmol) of solid 1,3,6,8-tetrakis(4-(methoxycarbonyl)phenyl)pyrene. The resultant suspension was strongly shaken under reflux overnight. Then, the solvents and water were added under vacuum to form a transparent yellow solution. Afterward, the clear yellow solution was shaken at room temperature for 2 h, and its pH-value was set to 1 using the concentrated HCl. The resulting yellow solid was filtered and washed with water several times. Then, the crude product was recrystallized from DMF and filtered off. Finally, chloroform was applied to wash the product, followed by vacuum drying that resulted in 0.49 g (91%) of pure H_4TBAPy product (Scheme S1).

Synthesis of $Zr_6(\mu_3\text{-}OH)_8(OH)_8(TBAPy)_2$ (NU-1000) $[Zr_6(\mu_3\text{-}OH)_4(\mu_3\text{-}O)_4(OH)_4(H_2O)_4(\mu_8\text{-}TBAPy)_2]$. According to the research design, 2.7 g of benzoic acid (C_6H_5COOH, 22 mmol) and 70 mg of zirconium chloride ($ZrCl_4$, 0.30 mmol) were blended in 8 mL of DMF (in a 6-dram vial) and treated by ultrasonication to dissolve. In the next stage, an oven was used to incubate the transparent solution at 80 °C for 1 h. Upon cooling to room temperature, 40 mg of H_4TBAPy ($C_{44}H_{26}O_8$, 0.06 mmol) was poured into the solution and the sonication was continued for 20 min. An oven was used to heat the yellow suspension at 120 °C for 48 h. Again, upon cooling of the suspension to room temperature, the filtration process was chosen to isolate the yellow poly-crystalline substance. Then, DMF was used to wash the product which was consequently treated with HCl as reported in Feng's study.[S2]

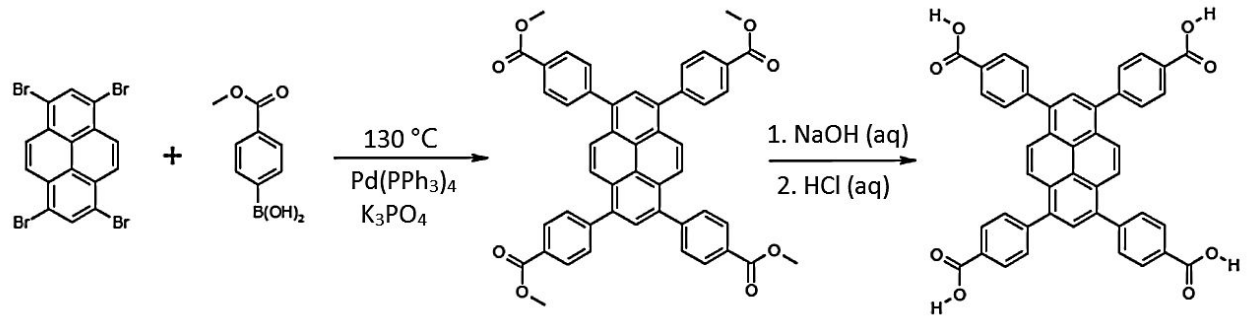

Scheme S1. Synthetic scheme for H_4TBAPy.

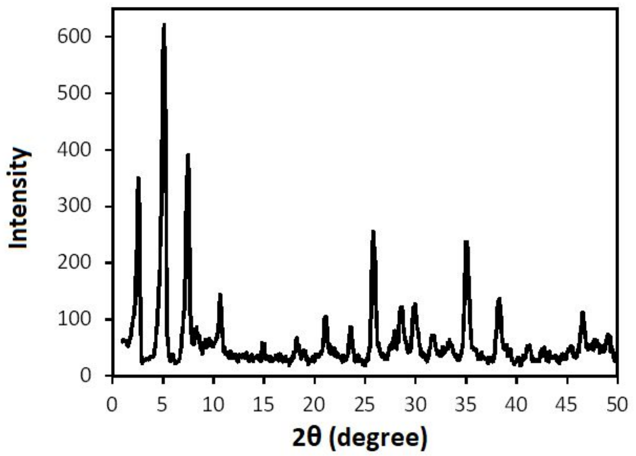

Figure S1. PXRD pattern of physically mixed sample containing SiW_{12} and NU-1000.

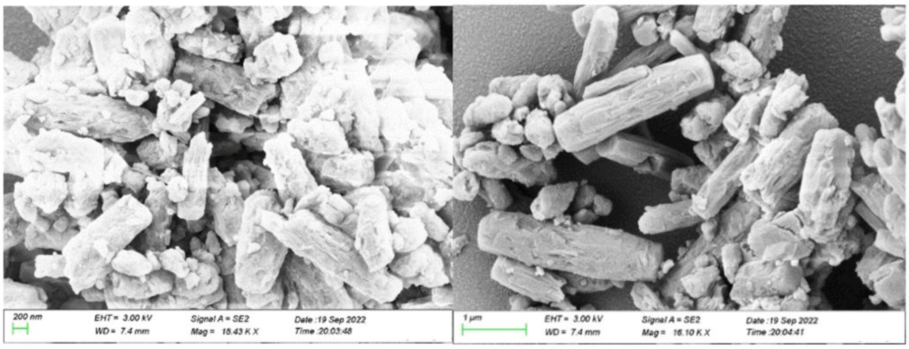

Figure S2. SEM images of physically mixed sample containing SiW_{12} and NU-1000.

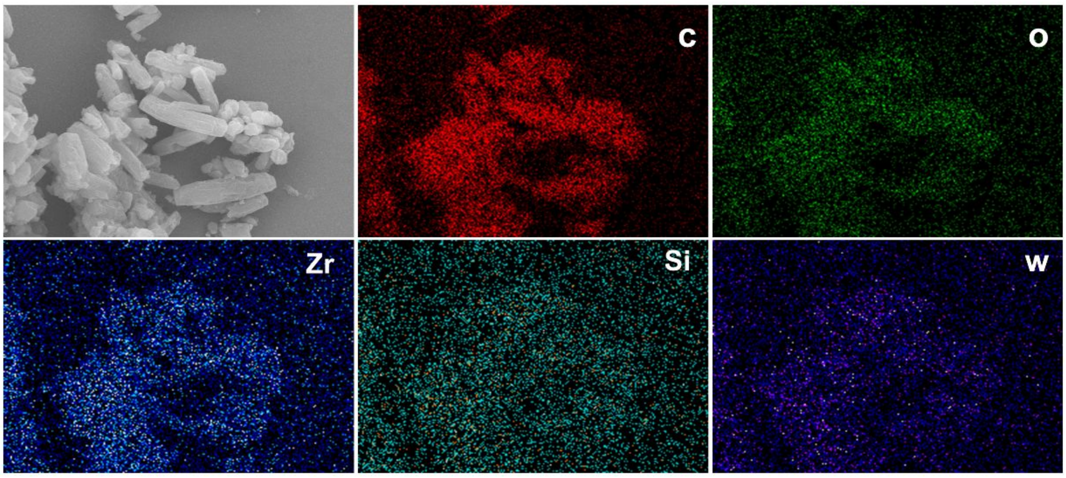

Figure S3. Elemental mapping of physically mixed sample containing SiW_{12} and NU-1000.

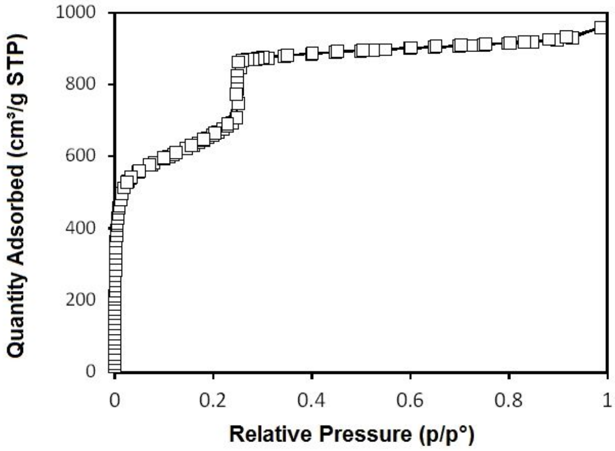

Figure S4. N_2 adsorption−desorption isotherm of a NU-1000 sample prepared following the protocol of composite formation in the absence of SiW_{12}.

Table S1. Nonlinear Optical Properties of POM-MOF in Comparison with Other Materials.

Materials	λ (nm)	$\boldsymbol{n_2}$	$\boldsymbol{\beta}$	Self-defoc./ Self-foc.*	2PA/SA/ RSA	Ref.
Porphyrin-POM	532	(1.3-16.8) $\times10^{-10}$ esu	(0.2-14.8) $\times10^{-5}$ esu	Self-defoc	RSA	(S3)
DiNTPP@Di-POM	532	1.65×10^{-9} esu	2.16×10^{-5} esu	Self-defoc	RSA	(S4)
Di-TPP@Di-POM	532	1.61×10^{-9} esu	1.8×10^{-5} esu	Self-defoc	RSA	(S4)
Gu-MOF	632.8	8.18×10^{-8} cm^2/W	7.47×10^{-3} cm/W	Self-defoc.	SA	(S5)
L5[1]	532	---	3.26×10^{-11} esu	---	TPA	(S6)
Zn-L5	532	---	40.17×10^{-11} esu	---	TPA	(S6)
Cd-L5	532	---	42.09×10^{-11} esu	---	SA	(S6)
ZIF-67[2]	1064	9.3×10^{-13} cm^2/W	0.85×10^{-9} cm/W	Self-defoc.	TPA	(S7)
ZIF-67	1342	7.2×10^{-13} cm^2/W	0.87×10^{-9} cm/W	Self-defoc	TPA	(S7)
Keplerate-type-POM[3]	532	(0.11-2.3)x10-9 esu	(1.02-1.37)$\times10^{-4}$ esu	Self-defoc	TPA	(S8)
H_2dcapp[4]	532	1.1×10^{-11} esu	---	Self-foc	TPA	(S9)
[Ag_{10}(dcapp)$_4$]·2(OH)·12H_2O	532	2.2×10^{-11} esu	---	Self-foc	TPA	(S9)
[Zn_4O(dcapp)$_3$]·6H_2O	532	1.2×10^{-11} esu	---	Self-foc	TPA	(S9)
[Hg_2(dcapp)$_2$]	532	1.3×10^{-11} esu	---	Self-foc	TPA	(S9)
ZIF-62(Zn,Co)	1030	-	3.37×10^{-9} cm/W	-	SA	(S10)
agZIF-62(Zn,Co)	1030	-	2.02×10^{-9} cm/W	-	SA	(S10)
POM-based $Fe_{10}P_4W_{32}$	532	---	15.4×10^{-6} esu	---	TPA	(S11)
POM-based Fe_8MoW_{18}	532	---	8.59×10^{-6} esu	---	TPA	(S11)
InPc-CPFs	532		3.0×10^{-8} cm/W	---	RSA	(S12)
FePc-CPFs	532	-	(3.4-4.47)$\times10^{-7}$ cm/W	-	RSA	(S12)
{(PEI=PSS=PAH)(PMo_{12}=bi-CoPc=PSS=PAH)$_6$}	532	30.6×10^{-12} esu	---	Self-defoc	SA	(S13)
{(PEI=PSS=PAH)(PEI=PSS)(bi-CoPc=PSS)$_6$}	532	8.88×10^{-12} esu	---	Self-foc	RSA	(S13)
NU-1000	532	22.26×10^{-8} cm^2/W	21.32×10^{-3} cm/W	Self-foc	TPA	This work
SiW_{12}@NU-1000	532	41.84×10^{-8} cm^2/W	23.64×10^{-3} cm/W	Self-foc	TPA	This work

[1] 3,7-bis(pyridine-4-yl)-10-(4–9(pyridine-4-yl) phenyl) − 10H-phenothiazine;

[2] Zeolitic Imidazolate Framework-67 (ZIF-67).

[3] Bu_4N-{Mo132}-polyoxometalate;

[4]2,6- dicarboxamido-2-pyridylpyridine

Reverse saturable absorption (RSA), saturable absorption (SA), two photon absorption (2PA).

Supporting References

(S1) Mondloch, J.E.; Bury, W.; Fairen-Jimenez, D.; Kwon, S.; DeMarco, E. J.; Weston, M. H.; Sarjeant, A. A.; Nguyen, S. T.; Stair, P. C.; Snurr, R. Q.; Farha, O. K.; Hupp, J. T. Vapor-phase metalation by atomic layer deposition in a metal–organic framework. *J. Am. Chem. Soc.* **2013**, *135*, 10294–10297.

(S2) Feng, D.; Gu, Z.-Y.; Li, J.-R.; Jiang, H.-L.; Wei, Z.; Zhou, H.-C. Zirconium-metalloporphyrin PCN-222: Mesoporous metal–organic frameworks with ultrahigh stability as biomimetic catalysts. *Angew. Chem. Int. Ed*. **2012**, *51*, 10307–10310.

(S3) Hassan, S.U.; Asif, H.M.; Zhou, Y.; Zhang, L.; Qu, N.; Li, J.; Shi, Z. "Closer is better and two is superior to one": Third-order optical nonlinearities of a family of porphyrin–anderson type polyoxometalate hybrid compounds. *J. Phys. Chem. C* **2016**, *120*, 27587–27599.

(S4) Asif, H.M.; Iqbal, A.; Zhou, Y.; Zhang, L.; Wang, T.; Ullah Farooqi, M.I.; Sun, R. Preparation, characterization and third order optical nonlinearities of looped covalently bonded Anderson-type polyoxometalate-porphyrin hybrids. *Dyes Pigm.* **2021**, *184*, 108758.

(S5) Siva, V.; Shameem, A.; Murugan, A.; Athimoolam, S.; Vinitha, G.; Bahadur, S. A. Structural, thermal and electro-optical properties of guanidine based metal-organic framework (MOF). *Chin. J. Phys*. **2020**, *68*, 764–777.

(S6) Jia, J.; Wang, J.; Li, M.; Gong, C.; Liang, G.; Song, Y.; She, Y. Phenothiazine metal-organic framework materials with excellent third-order nonlinear properties. *Dyes Pigm.* **2022**, *205*, 110398.

(S7) Pan, H.; Chu, H.; Wang, H.; Li, Y.; Zhao, S.; Li, G.; Li, D. Optical nonlinearity of zeolitic imidazolatef-67 in the near-infrared region. *Mater. Chem. Front*. **2020**, *4*, 2081–2088.

(S8) Zhou, Y.; Shi, Z.; Zhang, L.; Hassan, S.U.; Qu, N. Notable third-order optical nonlinearities of a Keplerate-type polyoxometalate in solution and in thin films of PMMA. *Appl. Phys. A* **2013**, *113*, 563–568.

(S9) Hou, H.; Wei, Y.; Song, Y.; Mi, L.; Tang, M.; Li, L.; Fan, Y. Metal ions play different roles in the third-order nonlinear optical properties of d^{10} metal–organic clusters. *Angew. Chem. Int. Ed*. **2005**, *44*, 6067–6074.

(S10) Mohamed, A.A.; Xiaofeng, L.; Yang, L.; Jinjun, R.; Jianrong, Q. Nonlinear-optical response in zeolitic imidazolate framework glass. *Inorg. Chem*. **2020**, *59*, 8380–8386.

(S11) Jiang, W.; Liu, X.-M.; Liu, J.; Shi, J.; Cao, J.-P.; Luo, X.-M.; You, W.-S.; Xu, Y. A novel polyoxometalate-based huge cluster $Fe_{10}P_4W_{32}$ exhibiting prominent electrocatalytic activity

for oxygen evolution reaction and third-order NLO properties *Chem. Commun*. **2019**, *55*, 9299–9302.

(S12) Wang, T.; Huang, W.; Sun, T.; Zhang, W.; Tang, W.; Yan, L.; Si, J.; Ma, H. Two-dimensional metal-polyphthalocyanine conjugated porous frameworks as promising optical limiting materials. *ACS Appl. Mater. Interfaces* **2020**, *12*, 46565–4657.

(S13) Xu, L.; Wang, E.; Li, Z.; Kurth, D.G.; Du, X.; Zhang, H.; Qin, C. Preparation and nonlinear optical properties of ultrathin composite films containing both a polyoxometalate anion and a binuclear phthalocyanine, *New J. Chem*. **2002**, *26*, 782–786.
